# The fourth-stage autoinfective larva of *Strongyloides stercoralis*: redescription and diagnostic implications

**DOI:** 10.1128/jcm.01021-24

**Published:** 2024-12-05

**Authors:** Huan Zhao, Anson V. Koehler, Cameron Truarn, Damien Bradford, David W. New, Rick Speare, Robin B. Gasser, Harsha Sheorey, Richard S. Bradbury

**Affiliations:** 1 School of Public Health and Tropical Medicine, College of Public Health, Medical and Veterinary Sciences, James Cook University 8001 , Townsville, Queensland, Australia; 2 Department of Veterinary Biosciences, Melbourne Veterinary School, Faculty of Science, The University of Melbourne 452644 , Parkville, Victoria, Australia; 3 PathWest Laboratory Medicine 56375 , Nedlands, Western Australia, Australia; 4 Microbiology Department, St. Vincents Hospital, Fitzroy, Victoria, Australia; Mayo Clinic Minnesota, Rochester, Minnesota, USA

**Keywords:** *Strongyloides stercoralis*, *Strongyloidiasis*, diagnosis, autoinfection, life cycle stages, larva

## Abstract

Human strongyloidiasis is often underdiagnosed or misdiagnosed, which can relate to a lack of knowledge or recognition of the importance of particular developmental/larval stages of *Strongyloides stercoralis* in making an accurate diagnosis using parasitological methods (a morphological approach or morphological features/characters). Here, we report the identification of *S. stercoralis* autoinfective fourth-stage larvae (L4a) in naturally infected humans, encountered in two clinical cases in Australia. These larvae were identified in sputum (Case 1) and bronchoalveolar lavage (Case 2) specimens by direct wet-mount microscopy. The L4a of *S. stercoralis* can be morphologically differentiated from autoinfective third-stage larvae by its conical and pointed tail and a relatively mature genital primordium with an enlarged genital rudiment and the formation of a vulva within cuticle layers. This study emphasizes the need to consider these morphological features of the L4a stage for an accurate diagnosis of *S. stercoralis* infection. A detailed morphological description of this stage is given to guide laboratory practitioners and researchers in the identification and differentiation of this unique but neglected life-cycle stage of *S. stercoralis*.

## INTRODUCTION

All nematodes have four larval stages (1). *Strongyloides stercoralis* (Bavay 1876) is a medically important parasitic nematode with a unique life cycle (2). This nematode has a remarkable ability to perpetuate within its host for decades and, when triggered by immunosuppression, can cause life-threatening systemic disease (3). The clinical diagnosis of *S. stercoralis* infection is challenging, and the microscopic detection and morphological identification of developmental stages of this parasite in feces or other biological specimens (e.g., sputum) remain a major diagnostic modality. Identification and differentiation of *S. stercoralis* from morphologically similar nematodes require the expertise of experienced morphologists. Recognizing and identifying morphological nuances in various stages of *S. stercoralis* are essential for an accurate and timely diagnosis of this neglected parasitic infection.

The life cycle of *S. stercoralis* is unusually complex. Parasitic adults are parthenogenetic females that reside in the intestinal mucosa. The eggs that they produce hatch within the crypts of Lieberkühn into rhabditiform larvae (L1r), which then migrate to the intestinal lumen and are passed in the feces (3, 4). The L1r molt into L2r and then, depending on environmental conditions, these L2r either develop into infective filariform larvae (L3i; homogonic cycle) or undergo four molts to become sexually reproducing free-living adult females and males, whose progeny are all L3i, which must infect a new host or die (heterogonic cycle) (3, 4). Environmental L3i from homogonic and heterogonic routes invade a host percutaneously to complete their life cycle (2–4).

*Strongyloides stercoralis* also undergoes an autoinfective cycle, wherein some L1r within the intestine transform into autoinfective filariform larvae (L3a). These larvae penetrate the host’s lower gut or perianal skin to re-establish infection (3–5). Autoinfection is believed to be the mechanism responsible for the chronicity of infection in hosts and for extensive multiplication during hyperinfection (3–5). The *in vivo* migratory routes of L3i and L3a involve both the cardio-pulmonary-esophageal pathway and random navigation to reach the small intestine, where they develop into a new generation of parasitic females (3–5).

Fourth-stage filariform larvae (L4) are known to occur in many human-parasitic skin-penetrating nematodes, including the hookworms (6), and have been observed in other *Strongyloides* species, such as *Strongyloides ratti* (7), *Strongyloides venezuelensis* (8), and *Strongyloides felis* (9). Remarkably, while four larval stages are recognized in the free-living life cycle of *S. stercoralis*, a fourth stage is not usually recognized in descriptions of the external infective and the autoinfective life cycles of this important human and animal parasite (4). Early experimental studies using canine (10) and non-human primate (11) models support the existence of at least one pre-adult, or “juvenile,” parasitic stage of *S. stercoralis*. These juvenile females were recognized as morphological intermediates between filariform L3 and parasitic adults and have been detected in the respiratory tract (10, 11). This information indicated that larval maturation can occur during pulmonary migration, supported by the recovery of adult worms from this site in experimental dogs (11–14) and in naturally infected humans (15, 16). However, until now, this pre-adult form has only been demonstrated experimentally, and its description was made based on an incorrect assumption at the time that parasitic males existed (10).

Diagnosis of “uncomplicated” strongyloidiasis typically relies on the identification of *S. stercoralis* L1r in the stool (17). Less commonly, filariform larvae may be found in respiratory specimens, such as bronchoalveolar lavage (BAL) fluid and sputum (17, 18). In clinical cases of hyperinfection, pulmonary involvement is more pronounced, and autoinfective filariform larvae may be abundant in these specimens (17). Thus, the detection and identification of autoinfective *S. stercoralis* larvae from clinical specimens are crucial for the early recognition of complicated strongyloidiasis.

Thus far, two filariform stages of *S. stercoralis*, i.e., L3i and L3a, have been morphologically characterized. They are distinguished from other major life stages of *S. stercoralis* by several body dimensions, most distinctly the esophagus and the tail (4) (Table 1). Morphological differences between L3i and L3a are subtle, yet discernible, with the former appearing longer and more slender (4) (Table 1).

**TABLE 1:** Morphological characteristics of the major life stages of *Strongyloides stercoralis* (8, 9, 18–21)

Life stage	Mean length (range) (µm)	Mean width^*a*^ (range) (µm)	Width^*a*^/ length (%)	Esophagus/ length (%)	Esophagus	Reproductive system	Tail
Parasitic female	2,420 (2,100–2,700)	37 (30–40)	1.5	23.8	Elongated cylindrical (filariform) esophagus	Straight reflected ovary; uteri short, usually containing no more than six eggs; vulva about two-thirds body length from anterior end, with pair of prominent muscles surrounding transverse opening; seminal receptacles absent	Narrowly tapered to a cone-shaped tail
Free-living female	1,140 (920–1,700)	62 (52–85)	5.4	12.6	Rhabditoid esophagus	Didelphic with opposed equal uteri and reflected ovaries; vulva near the middle of the body; seminal receptacles present	Gradually tapered to a finely pointed tail
Free-living male	900 (810–1,000)	43 (40–50)	4.8	13.1	Rhabditoid esophagus	Straight tubule structure; two small sickle-shaped spicules and a single gubernaculum	Gradually tapered to a finely pointed tail; often curved ventrally.
Rhabditiform (L1r) larvae	210 (180–240)	14.5 (14–15)	6.9	30	Rhabditoid esophagus	Lateral rhomboid genital primordium halfway down the larval body	Gradually tapered to a finely pointed tail
Infective filariform (L3i) larvae	563 (490–630)	15.8 (15–16)	2.8	43	Elongated cylindrical (filariform) esophagus	A small genital rudiment visible at about the midpoint of the intestine	Truncated notched tail
Autoinfective third-stage filariform (L3a) larvae	269 (234–317)	11 (10–13)	4.1	42–52	Elongated cylindrical (filariform) esophagus	A small genital rudiment visible at about the midpoint of the intestine	Truncated notched tail
Autoinfective fourth-stage filariform (L4a) larvae^*b*^	nd^*c*^	nd	3.7–5.2	37–46	Elongated cylindrical (filariform) esophagus	Elongated genital rudiment midway of the intestine; vulva formed as a transverse slit at the midpoint of the intestine but has an overlying layer of cuticle	Narrowly tapered to a point or to a cone-shaped tail

^*a*^ At the widest point.

^*b*^ Data derived from the present study.

^*c*^ nd, no data.

This study presents an unusual autoinfective stage of *S. stercoralis* identified in respiratory tract specimens of two patients in Australia. Our findings indicate the need to expand diagnostic parameters to include this clinically important yet neglected filariform stage of *S. stercoralis*.

## MATERIALS AND METHODS

### Cases

This is a retrospective study of diagnostic data for two clinical cases of disseminated strongyloidiasis occurring in Australia within the past 10 years. The patients were from South East Asia and East Africa, respectively, and had resided in Australia for a number of decades. They reported no recent travel history to endemic countries prior to admission.

As part of the routine investigation for respiratory infections, a sputum specimen was collected from Patient 1, and bronchoscopy was performed to obtain BAL fluid from Patient 2. Upon collection, respiratory specimens were sent to a hospital microbiology laboratory within 4 hours for microscopic analysis.

### Laboratory processing and microscopy

Specimens were processed immediately (within 1 hour) upon arrival. The BAL specimen was centrifuged at 500 × *g* for 20 minutes, and the sputum specimen was centrifuged at 500 × *g* for 10 minutes. Supernatants were discarded, and two wet mount slides were prepared from each specimen and examined by microscopy at 100- and 400-times magnification. Measurements were not taken in this routine pathology setting, but the identified larvae were photographed, and the percentage ratios of body length, maximum width, and esophagus length were determined by examination of those photographs.

### Molecular and phylogenetic analyses

The remaining sputum (*n* = 1) and BAL (*n* = 1) specimens underwent DNA extraction by first digesting with proteinase K (10 µg/µL) (Promega, USA) and lysis buffer for 2 hours at 56°C and then using the Promega DNA Clean Up Kit, following the manufacturer’s protocol. PCR of the mitochondrial cytochrome *c* oxidase subunit I (*cox*1) gene was performed as previously described (22, 23), and the amplicons were subjected to Sanger sequencing. Sequence data were BLASTN searched against the GenBank database containing all available nematode sequences. These sequences were placed in a maximum likelihood tree generated in MEGA11 (24) along with other sequences of *Strongyloides* and *Necator americanus* as an outgroup.

## RESULTS

### Microscopic findings

**Fig 1:**
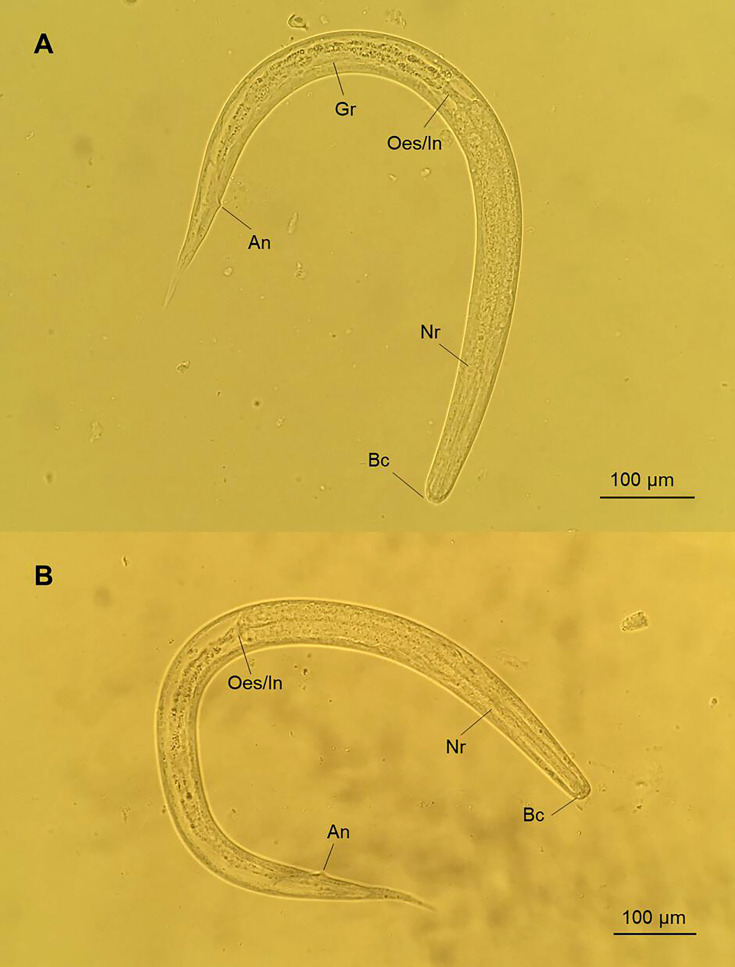
*Strongyloides stercoralis* autoinfective fourth-stage larvae (**A and B**) recovered from the sputum of Patient 1. An, anus; Bc, buccal cavity; Gr, genital rudiment; Nr, nerve ring; Oes/In, esophageal-intestinal junction.

**Fig 2:**
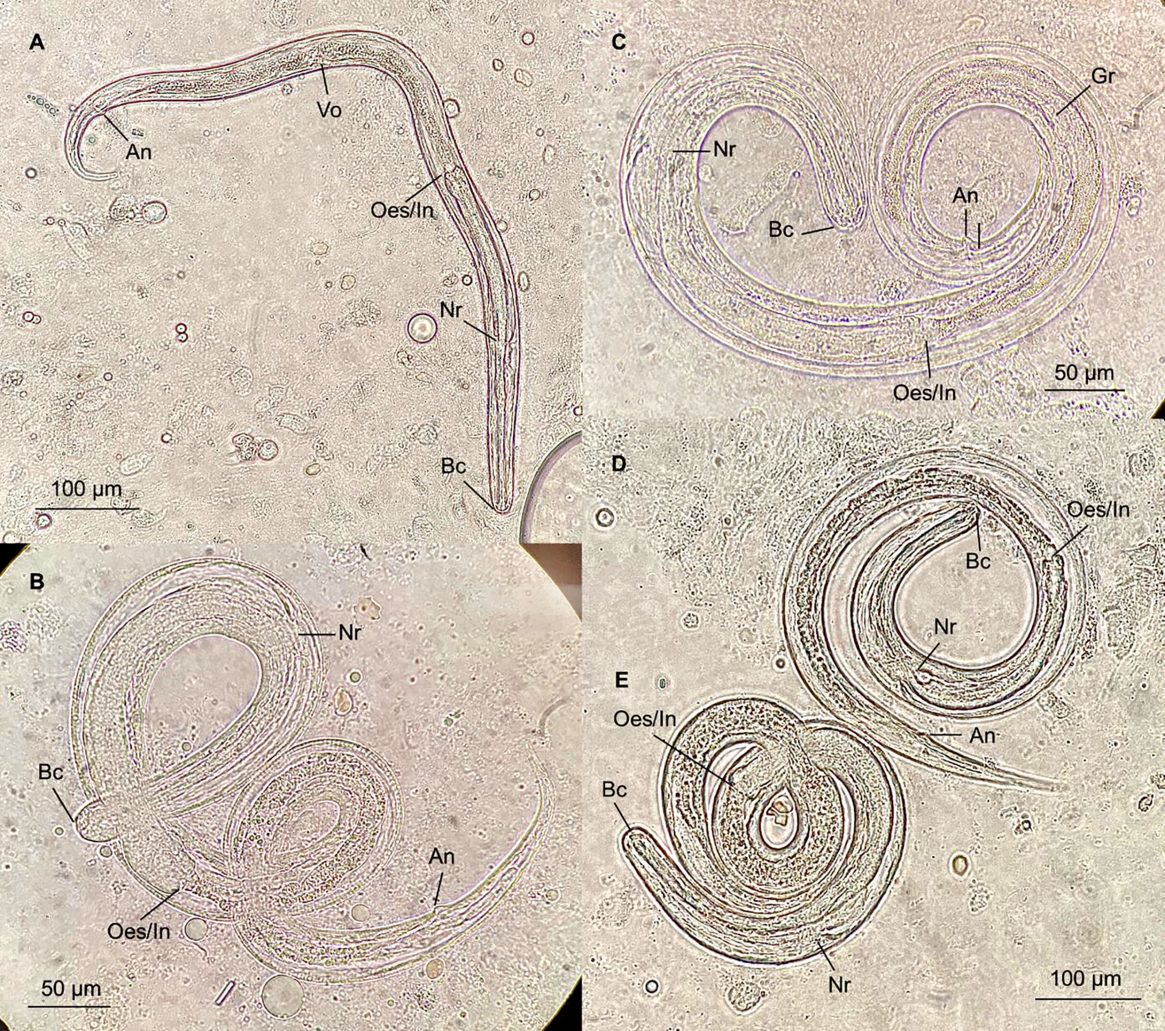
*Strongyloides stercoralis* autoinfective fourth-stage larvae (**A through E**) recovered from the BAL fluid of Patient 2. An, anus; Bc, buccal cavity; Gr, genital rudiment; Nr, nerve ring; Oes/In, esophageal-intestinal junction; Vo, vaginal opening.

Multiple larvae were recovered from the respiratory specimens (Fig. 1 and 2). These larvae closely resembled the L3a stage of *S. stercoralis* in several aspects. Specifically, they appeared slender, with a maximum width-to-length ratio of 3.7%–5.2% (Table 2). The buccal cavity was shallow, with a small pore-like mouth structure. The esophagus was cylindrical, filariform, extending 37%–46% of the body length (Table 2). The anus was evident sub-terminally, with small lip-like swellings along the posterior edge of the transverse opening.

**TABLE 2:** Morphological features of *Strongyloides stercoralis* autoinfective fourth-stage larvae

Case	Width^*a*^/ length (%)^*b*^	Esophagus/ length (%)^*b*^	Esophagus	Excretory system	Reproductive system	Cuticle	Tail
Case 1	4.9–5.2	46	Filariform esophagus	Anus subterminal, with small lip-like swelling along the posterior edge of transverse opening	Elongated genital rudiment midway of the intestine	Finely striated cuticle	Narrowly tapered to a point
Case 2	3.7–4.6	37–46	Filariform esophagus	Anus subterminal, with small lip-like swelling along the posterior edge of transverse opening	Elongated genital rudiment midway of the intestine; vulva formed as a transverse slit at the midpoint of the intestine but has an overlying layer of cuticle	Finely striated cuticle	Narrowly tapered to a cone-shaped tail

^*a*^ At the widest point.

^*b*^ Average of five measurements.

However, the reproductive systems of these larvae appeared to be more mature than those of the L3a stage, but not as developed as the parasitic female. Specifically, an enlarged genital rudiment was visible around the midpoint of the intestine. In one larva, the vulva formed as a transverse slit but with an overlying layer of the cuticle (Fig. 2A). This larva had a notably lower width-to-length ratio (3.7%), and its esophagus was comparatively shorter relative to the body length (37%) than L3a. Ovary or uteri structures were inconspicuous. Moreover, the tails of these larvae were not notched but narrowly tapered to a cone shape or a point, resembling those observed in the parasitic adult stage (Fig. 1 and 2).

**Fig 3:**
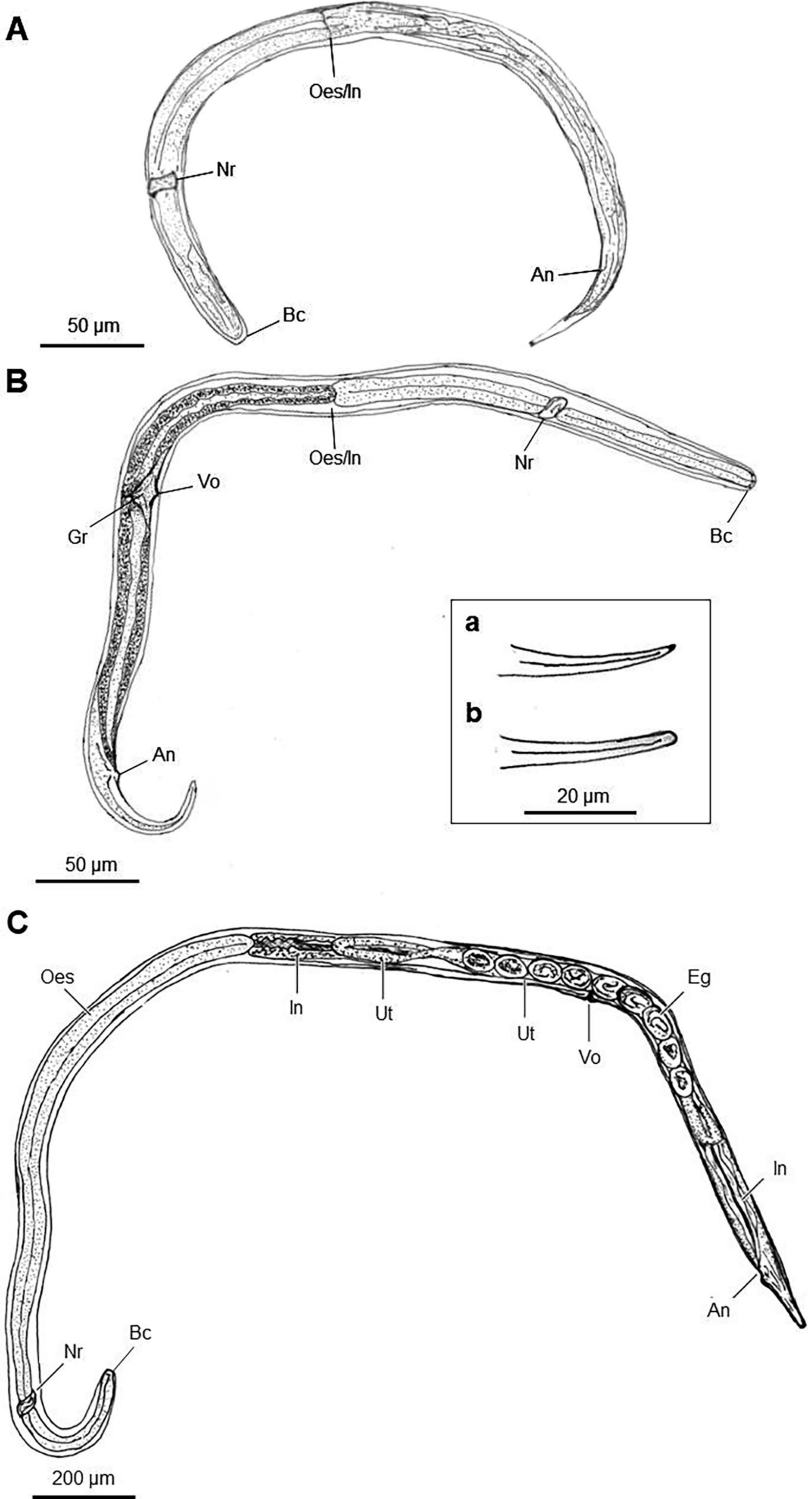
Anatomical drawings of the third-stage autoinfective larva of *Strongyloides stercoralis* (**A**), fourth-stage autoinfective larva (**B**), and parasitic adult (**C**) (4). The tail of L4a is either pointed (a) or cone-shaped (b). An, anus; Bc, buccal cavity; Eg, eggs; Gr, genital rudiment; In, intestine; Nr, nerve ring; Oes, esophagus; Oes/In, esophageal-intestinal junction; Ut, uterus; Vo, vaginal opening.

These larvae appear to represent a transitional form between the filariform L3 stage and the parasitic adult stage of *S. stercoralis* (Fig. 3). Specifically, these recovered larvae are morphologically consistent with the *S. stercoralis* autoinfective fourth-stage filariform larvae described by Faust in 1933 (10).

Several L3a were identified from the BAL specimen (Fig. S1). No other parasite eggs, larvae, cysts, or trophozoites were identified in either of the two specimens by microscopy.

### Molecular findings

**Fig 4:**
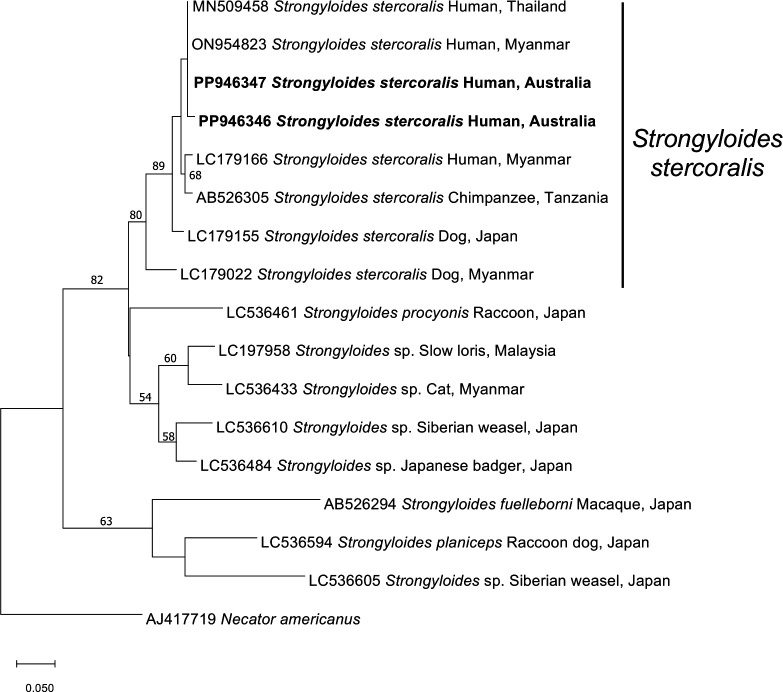
Maximum likelihood tree based on *cox*1 sequences generated in this study (in bold) and those published in the literature.

Sequencing confirmed that the larvae belonged to *S. stercoralis* (GenBank accession numbers PP946346 and PP946347), both having a 99.62% (964 bp) and 99.68% (393 bp) identity, respectively, to known *S. stercoralis* sequences (GenBank accession numbers MN509458 and ON954823). A maximum likelihood phylogenetic tree placed these sequences within the *S. stercoralis* clade (Fig. 4).

## DISCUSSION

The L4a stage has until now been neglected in the scientific and clinical literature, and its detection in these two clinical cases caused confusion even among the experienced morphologists/parasitologists authoring this paper when it was first encountered. The presence of thin and tapered nematode larvae in sputum and BAL fluid indicated *S. stercoralis*, but the unfamiliar morphology meant that this diagnosis could not be confirmed in the first instance. DNA sequencing of well-known genetic markers confirmed the species identity, and further investigation of the literature identified these as the neglected juvenile or pre-adult stage of *S. stercoralis*. Here, we identify these as juvenile or pre-adult forms as the missing fourth-stage autoinfective larva (L4a) of *S. stercoralis*.

Pre-adult parasitic females of *S. stercoralis* have been only documented twice in the literature (10, 11). Faust (10) in 1933 described two premature parasitic stages of *S. stercoralis*, referred to as “the preadolescent female” and “the adolescent female,” recovered from the lung of experimentally infected dogs. Marked developments in these two stages included the enlargement of genital primordia and the formation of a vulva opening in some cases. These findings accord with our observations of *S. stercoralis* L4a in the present case series. However, Faust (10) noted that the notch of the tail persisted in juvenile females, contrasting our finding of the conical or pointed tail morphology. Additionally, despite an increase in body length, the esophagus of pre-adult females was shorter compared with the L3i stage, while this difference was inconspicuous in our study. Faust’s work was conducted in an era when parasitic *S. stercoralis* males were believed to exist. It is possible that his description reflected a combination of parasitic and free-living stages. The only other study reporting *S. stercoralis* parasitic pre-adults was that of Mati et al. in 2014 (11), who used the marmoset experimental model. Faust’s criteria (10) were followed for larval identification. It was noted that the esophagus-to-body length ratio of the identified worms was intermediate between the ratios seen in L3i and adult females, consistent with our findings for some L4a. No further morphological characterization was made in the study by Mati et al. (11).

L4 filariform larvae have been previously reported in the life cycle of *S. felis*, a larviparous *Strongyloides* species infecting cats. According to Speare (9), this larval stage was characterized by a non-notched tail morphology and conspicuous reproductive maturation, with the vulva forming as a transverse slit within layers of cuticle. These findings closely mirror our findings for *S. stercoralis* L4a. Phylogenetically, *S. felis* is closely related to *S. stercoralis* (25), and, therefore, it is very plausible that the two species share morphological similarities at different life stages.

One limitation of this work is the lack of morphometric data for the *S. stercoralis* larvae identified. Consequently, we were unable to experimentally assess the developmental progression of parasitic stages based on size changes. Despite this issue, our morphological characterization of the L4a stage has substantial diagnostic value. Established criteria for identifying autoinfective larvae rely heavily on the tail morphology. While the characteristic notched tail of the L3a stage differentiates it from other nematodes infecting humans, it should not be the only consideration for the diagnosis of hyperinfective strongyloidiasis. The present study indicates the need for including such diagnostic criteria for this neglected L4a stage of *S. stercoralis*. Future work is needed for more detailed characterization of this larval stage.

This study was based on the observation of two clinical cases. Given this limited data set, these descriptions of the L4a stage of *S. stercoralis* should be considered preliminary and would benefit from further evidence in broader clinical contexts. The clinical data available for these cases were sparse. While larvae were detected during the initial diagnosis of strongyloidiasis, the impact of anthelmintic therapies on the occurrence of this stage is unclear. Future research is needed to fully understand the implications of the L4a stage in respiratory specimens on the severity, progression, and treatment outcomes of strongyloidiasis.

It is important for the clinical parasitology community to recognize and identify rare diagnostic stages of parasites. In these cases, identification of the infecting agents as *S. stercoralis* was delayed due to the very unusual morphology observed. Early involvement of infectious disease specialists is essential in any suspected case of *Strongyloides* hyperinfection or systemic disease. Given the risk of disseminated strongyloidiasis, particularly in immunocompromised patients, empiric treatment may be recommended before diagnostic confirmation when clinical suspicion is high. This approach can be crucial in preventing severe complications and should be guided by the overall clinical presentation, risk factors, and symptom severity.

The complexities in diagnosing *S. stercoralis* infection highlight the ongoing need to maintain laboratory and morphological skills, especially in light of the global progressive loss of expertise in morphology-based parasitic diagnostics (26). Training programs should emphasize the differentiation of all *S. stercoralis* life stages from other human-infecting nematodes, such as hookworms. To address diagnostic challenges, the integration of targeted molecular testing, such as *Strongyloides* PCR and sequencing (27), should complement traditional methods in clinical diagnostic practice. Furthermore, proper specimen processing, such as centrifuging BAL specimens to concentrate any parasitic or other diagnostic elements present, is essential to prevent false-negative results.

### Conclusion

We report and redescribe the “forgotten” juvenile form of the parasitic female of *S. stercoralis*, observed in two clinical cases in Australia, and identify this form as the fourth-stage autoinfective larva. This developmental stage is a morphological intermediate between the L3a and the parasitic adult stage of *S. stercoralis*. It is important that clinical and veterinary microbiologists, as well as parasitologists, be aware of the morphological features of this stage in order to avoid diagnostic confusion and delayed diagnosis and treatment when this stage is encountered in extra-intestinal specimens from human or animal patients suffering from *Strongyloides* hyperinfection or systemic strongyloidiasis.

## SUPPLEMENTAL MATERIAL

Figure S1 jcm.01021-24-s0001.docx *Strongyloides stercoralis* autoinfective third-stage larva recovered from the bronchoalveolar lavage (BAL) fluid of patient 2.

ASM does not own the copyrights to Supplemental Material that may be linked to, or accessed through, an article. The authors have granted ASM a non-exclusive, world-wide license to publish the Supplemental Material files. Please contact the corresponding author directly for reuse.
