# Supplementary material for: The fourth-stage autoinfective larva of Strongyloides stercoralis: redescription and diagnostic implications
Source: J Clin Microbiol. 2024 Dec 5;63(1):e01021-24. doi: 10.1128/jcm.01021-24 (PMC11784425; doi:10.1128/jcm.01021-24)

Supplementary Figure 1. *Strongyloides stercoralis* autoinfective third-stage larva recovered from the bronchoalveolar lavage (BAL) fluid of Patient 2. The notched tail is highlighted. Bc, buccal cavity; Nr, nerve ring; Oes/In, oesophageal-intestinal junction.


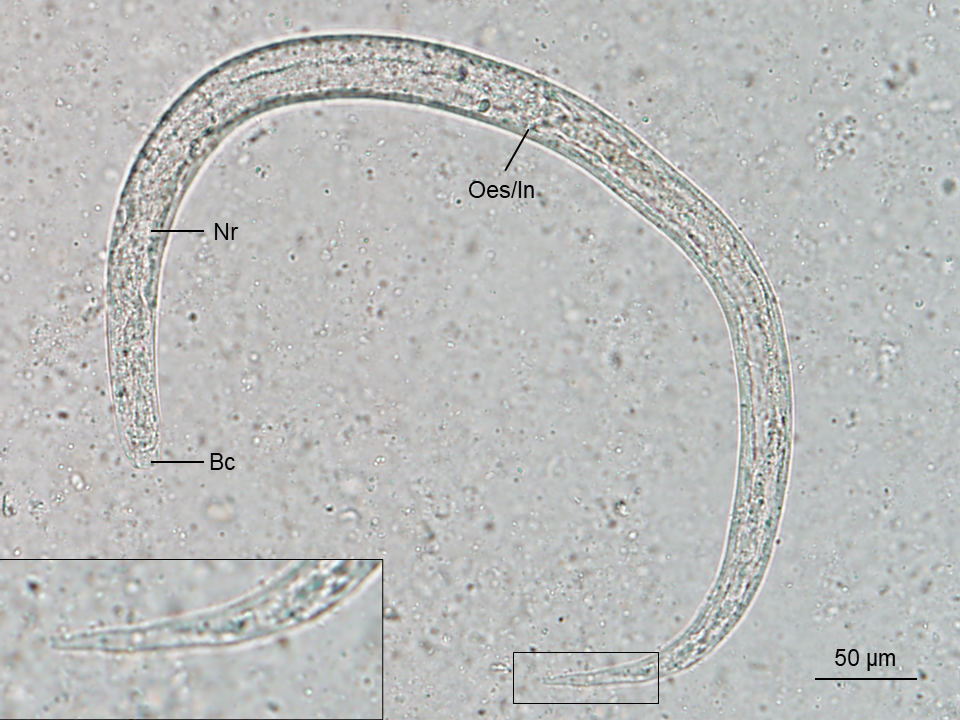

Supplement: Figure S1 — Strongyloides stercoralis autoinfective third-stage larva recovered from the bronchoalveolar lavage (BAL) fluid of patient 2. [file jcm.01021-24-s0001.docx]
